# Hand hygiene knowledge and attitude of medical students in western Saudi Arabia

**DOI:** 10.7717/peerj.6823

**Published:** 2019-04-25

**Authors:** Marwan A. Bakarman, Mukhtiar Baig, Ahmad A. Malik, Zohair J. Gazzaz, Mostafa M. Mostafa, Mohamed A. Zayed, Abdulaziz S. Balubaid, Ahmed K. Alzahrani

**Affiliations:** 1 Department of Family and Community Medicine, Faculty of Medicine, Rabigh, King Abdulaziz University, Jeddah, Saudi Arabia; 2 Department of Clinical Biochemistry, Faculty of Medicine, Rabigh, King Abdulaziz University, Jeddah, Saudi Arabia; 3 Department of Medicine, Faculty of Medicine, Rabigh, King Abdulaziz University, Jeddah, Saudi Arabia; 4 Department of Physiology, Faculty of Medicine, Rabigh, King Abdulaziz University, Jeddah, Saudi Arabia; 5 Sixth-year Medical Students, Faculty of Medicine, Rabigh, King Abdulaziz University, Jeddah, Saudi Arabia; 6 University Institute of Public Health, The University of Lahore, Pakistan

**Keywords:** WHO Questionnaire, Medical students, Knowledge, Attitude, Hand hygiene

## Abstract

**Background:**

The practice of hand hygiene (HH) has prime importance among Health Care Professionals (HCPs) and non-compliance could cause adverse consequences. By keeping the importance of HH in mind, this study aims at investigating the knowledge and attitudes towards HH among medical students and interns at King Abdulaziz University (KAU), Jeddah, Saudi Arabia (SA).

**Methods:**

The study includes 453 medical students and interns (189 males & 264 females). This study was completed in three months; from September to November 2017. The World Health Organization (WHO) HH questionnaire was used and data were analyzed on SPSS-21.

**Results:**

Two-third of the participants 292 (64.2%) had formal training in HH in the last three years. Almost half of the participants 254 (56.1%) had correct knowledge regarding the major course of transmission of potentially detrimental microbes among patients in the healthcare premises. Just 124 (27.4%) of the respondents had the correct knowledge about the most common basis of germs accountable for healthcare-related infections. Females had significantly better knowledge than males regarding the type of HH technique needed before palpation of the abdomen (177(54%) Vs. 151(46%); *P* < 0.002), before an injection (175(54.5%) Vs. 146(45.5%); *P* < 0.007), after emptying a bedpan (207(64.7%) Vs. 113(35.3%); *P* < 0.001), following discarding examination gloves (256(60.4% Vs. 168(39.6%); *P* < 0.001] and after exposure to blood (200(64.1%) Vs. 112(35.%); *P* < 0.001). Female participants had better knowledge than males regarding the type of HH actions. Females also had a significantly better attitude towards the importance of HH than males (240(62.5%) Vs. 144(37.5%); *P* < 0.001).

**Conclusion:**

The majority of the participants’ knowledge regarding HH was not up to the mark; however, female students had better knowledge compared to male students. The medical students and interns’ knowledge and positive attitude towards HH can play a pivotal role in preventing HCPs associated infections and it would overall decrease the infection-related burden on the hospital and governmental budgets. It is suggested that multi-dimensional plans are required to change this low compliance to a higher rate.

## Introduction

Healthcare-associated infections are considered a big problem affecting healthcare systems. They are associated with prolonged hospitalization, higher resistance to antimicrobial drugs, higher morbidity and mortality rates, and higher costs of healthcare services (Huang et al., 2013). Hand hygiene is a simple, economical and practical action that lessens the prevalence of healthcare-related infections in all healthcare centers (Kelcíkova, Skodova & Straka, 2012).

Hand hygiene is reported in the guidelines of various international organizations as a preventive issue to reduce healthcare-associated infections (HAIs) (WHO, 2009; Boyce et al., 2002).

The World Health Organization (WHO) has presented strategies to help improve awareness and training of HH among all workers of the healthcare profession (WHO, 2009). These guidelines have been widely used by the hospitals, but are not significantly emphasized in the medical curriculum (Thakker & Jadhav, 2015).

Globally, millions of patients suffer from infections that are transferred by healthcare providers (HCPs) (Pourakbari et al., 2012; Melaku et al., 2012). However, a good number of these infections can be stopped by implementing the simple technique of HH (WHO, 2010). This literature indicates the various proportion of knowledge and practices of HH among medical students and other healthcare workers (Sai et al., 2017; Modi et al., 2017). It also shows that there is a dearth of compliance with HH. Therefore, incessant hard work is needed to develop efficient and maintainable plans (Sai et al., 2017).

Healthcare providers could be the main source of germs spread if their hands are not properly washed (Boyce et al., 2009). All healthcare workers must know the HH techniques correctly and should be able to perform it accurately (Modi et al., 2017).

Medical students play an important role in healthcare teams as they frequently come in contact with patients during their clinical rotations. They could be a source of infection in their passage in different hospital sectors as neonatal rooms and operating theatres. Therefore, it’s very important for them to be aware of guidelines for infection control especially HH to limit the spread of infections (Herbert et al., 2013).

The study aims to measure the knowledge, and attitudes towards HH among medical students and interns at King Abdulaziz University, Jeddah. Our study result would help the authorities to take corrective and adaptive measures for improving the HH knowledge and compliance in not only our medical school but also in other schools of the SA.

## Materials & Methods

The present cross-sectional survey was done at the Faculty of Medicine, Rabigh, KAU, Jeddah, SA. The study included 453 students from the Rabigh and Jeddah campuses. The Research Ethics Committee of Faculty of Medicine, Rabigh, King Abdulaziz University, Jeddah, gave approval (Approval No. FMR-02-39-H) for this study and all participants’ verbal consent was sought. This study was completed in three months; from September to November, 2017. The second, third, fourth, fifth and sixth years medical students and interns were included in this survey and “WHO’s hand hygiene knowledge questionnaire for healthcare workers” (WHO) was used to assess participants knowledge while attitude and practice questions were taken from another study (Nair et al., 2014). We also collected the data about the respondents’ characteristics such as age, year of study, gender, study campus either Rabigh or Jeddah. The questionnaire comprised of a variety of questions including: “questions about training or seminars attended about hand hygiene in the past few months, awareness of the risk that they can be the source of cross-contamination in the hospital, and awareness of the effectiveness of hand hygiene to prevent HAIs.” Moreover, it also investigated the participants’ knowledge regarding “possible routes of contamination in healthcare facilities, most frequent sources of germs that can cause HAIs, hand hygiene actions that prevent transmission of germs to patients and healthcare workers, hand hygiene using soap and water and by alcohol-based hand rub, and actions that can increase colonization of germs in the hands.” Also included in the questionnaire are questions with “yes” or “no” answers, and multiple choice questions. The instrument was evaluated by the penal of experts which included a physician, and medical educationist, for the effectiveness of the questions and correct language of the questionnaire (content validity). For the reliability of the questionnaire, it was pretested on 50 students and calculated Cronbach’s alpha was .84. The WHO questionnaire had included questions with the “yes” or “no” answers, and multiple-choice questions.

We invited 600 medical students and interns, but 453 consented and returned their completely filled proforma. The sample size was calculated by using a sample size calculator at Statistical Solutions (https://www.statisticssolutions.com/directory-of-statistical-analyses/). The confidence level was 95%, a margin of error was 5%, population size was taken as 2200 by assuming the 50% of participants that had good HH knowledge and the power of the study was 80%. The calculated sample size was 327, but by considering 10% of students’ non-response rates, more students were invited to participate.

The Faculty of Medicine Rabigh, is comparatively a new faculty located near the red seashore at the Rabigh town that is about 160 Km away from the Jeddah city (Imran et al., 2016). The healthcare system of SA is controlled by the Ministry of Health (MOH) and all healthcare facilities to the citizens of SA are provided free of charge at the public sector hospitals and MOH has an extensive setup of primary care centers all over the Kingdom (Dhafar et al., 2015). Saudi government spent a significant proportion of their annual budget on health and education.

Students were requested to participate via personal contacts, via email communication and class announcements. Students were assured strict confidentiality of the data and they were assured that their identification would not be disclosed.

Statistical analysis was performed using SPSS (version 21) software. Data is represented as frequency and percentages. The comparison of knowledge between male and female students was calculated using the chi-square test. The *P* < 0.05 was taken as significant.

## Results

A total of 453/600 participated in this survey, and the participation rate was 75.5%. Out of 453 participants, 264 (58.3%) were females, and 189 (41.7%) were males. The mean age of the participants was 22.33 ± 1.79 years (Table 1).

**Table 1:** General characteristic of the participants.

**Variables**	***N* (%)**
**Age** (yrs) (Mean ± SD)	22.33 ± 1.79
**Gender**	
Male	189(41.7)
Female	264(58.3)
**Year of study**	
2nd	47(10.4)
3rd	87(19.2)
4th	56(12.4)
5th	57(12.6)
6th	151(33.3)
Interns	55(12.1)

**Notes.**

*N* number of study participants % percentage

About two-thirds of the participants 291(64.2%) had formal training in HH in the last three years and 303 (66.9%) of them were routinely using an alcohol-based hand rubbing method for HH. Almost half of the participants 254 (56.1%) had correct knowledge regarding the major cause of transmission of potentially detrimental microbes among patients in the healthcare premises. Surprisingly, just 124 (27.4%) of the respondents had the correct knowledge about the most common basis of germs accountable for healthcare-related infections (Table 2A).

**Table 2:** Participants’ response to questions and gender-wise comparison regarding hand hygiene.

**Questions**	**Yes*N* (%)**	**No*N* (%)**	**Female*N* (%)**	**Male*N* (%)**	***p*-value**
**A.**					
Formal training in HH in the last three years	Yes = 291(64.2)	No = 162(35.8)	185(63.6)	106(36.4)	0.002
Routinely use an alcohol-based hand rub for HH	Yes = 303(66.9)	No = 150(33.1)	192(63.4)	111(36.6)	0.001
**The main route of cross-transmission of potentially harmful germs between patients in a health-care facility**					
a. Health-care workers’ hands when not clean **(correct)**	254(56.1)		152(59.8)	102(40.2)	0.25
b. Air circulating in the hospital	38(8.4)				
c. Patients’ exposure to colonized surfaces (i.e., beds, chairs, tables, floors)	121(26.7)				
d. Sharing non-invasive objects (i.e., stethoscopes, pressure cuffs, etc.) between patients	40(8.8)				
**The most frequent source of germs responsible for health care-associated infections**					
a. The hospital’s water system	00				
b. The hospital air	00				
c. Germs already present on or within the patient **(correct)**	124(27.4)		65(52.4)	59(47.6)	0.07
d. The hospital environment (surfaces)	329(72.6)				
**The following hand hygiene actions prevents transmission of germs to the patient**					
a. Before touching a patient **(yes)**	Yes 404(89.2)	No 49(10.8)	246(60.9)	158(39.1)	0.001
b. Immediately after a risk of body fluid exposure **(yes)**	337(74.4)	116(25.6)	207(61.4)	130(38.6)	0.014
c. After exposure to the immediate surroundings of a patient **(no)**	350(77.3)	103(22.7)	210(60)	140(40)	0.105
d. Immediately before a clean/aseptic procedure **(yes)**	410(90.5)	43(9.5)	242(59)	168(41)	0.202
**Which of the following hand hygiene actions prevents transmission of germs to the health-care worker?**					
a. After touching a patient **(yes)**	402(88.7)	51(11.3)	231(57.5)	171(42.5)	0.202
b. Immediately after a risk of body fluid exposure **(yes)**	379(83.7)	74(16.3)	232(61.2)	147(38.8)	0.003
c. After exposure to the immediate surroundings of a patient **(yes)**	387(85.4)	66(14.6)	233(60.2)	154(39.8)	0.031
d. Immediately before a clean/aseptic procedure **(no)**	344(75.9)	109(24.1)	203(59)	141(41)	0.325
**B.**					
**Which of the following statements on alcohol-based hand rub and hand washing with soap and water are true?**					
a. Hand rubbing is more rapid for hand cleansing than hand washing **(true)**	True 251(55.4)	False 202(44.6)	127(50.6)	124(49.4)	<0.001
b. Hand rubbing causes skin dryness more than hand washing **(false)**	223(49.2)	230(50.8)	128(57.4)	95(42.6)	0.390
c. Hand rubbing is more effective against germs than hand washing **(false)**	154(34)	299(66)	203(67.9)	96(32.1)	<0.001
d. Hand washing and hand rubbing are recommended to be performed in sequence **(false)**	320(70.6)	133(29.4)	86(64.7)	47(35.3)	0.047
** What is the minimal time needed for alcohol-based hand rub to kill most germs on your hands?**					
a. 1 min	122(26.9)				
b. 20 s **(true)**	135(29.8)		77(57)	58(43)	0.402
c. 10 s	149(32.9)				
d. 3 s	47(10.4)				
**Which type of hand hygiene method is required in the following situations?a. Rubbing b. Washing c. None**					
Before palpation of the abdomen (rubbing)	328(72.4)		177(54)	151(46)	0.002
Before giving an injection (rubbing)	321(70.9)		175(54.5)	146(45.5)	0.007
After emptying a bed pan (washing)	320(70.6)		207(64.7)	113(35.3)	<0.001
After removing examination gloves (rubbing/washing)	424(93.6)		256(60.4)	168(39.6)	0.001
After making a patient’s bed (rubbing)	210(46.4)		119(56.7)	91(43.3)	0.291
After visible exposure to blood (washing)	312(68.9)		200(64.1)	112(35.9)	<0.001
**Which of the following should be avoided, as associated with increased likelihood of colonisation of hands with harmful germs?**					
a. Wearing jewelry **(yes)**	Yes 333(73.5)	No 120(26.5)	202(60.7)	131(39.3)	0.055
b. Damaged skin **(yes)**	336(74.2)	117(25.8)	189(56.3)	147(43.8)	0.084
c. Artificial fingernails **(yes)**	389(85.9)	64(14.1)	54(57.4)	40(42.6)	0.472
d. Regular use of a hand cream **(no)**	94(20.8)	359(79.2)	244(62.7)	145(37.3)	<0.001
Sometimes I miss out hand hygiene simply because I forget it (practice)	263(58.1)	190(41.9)	146(55.5)	117(44.5)	.095
I feel guilty if I omit hand hygiene (attitude)	384(84.8)	69(15.2)	240(62.5)	144(37.5)	<0.001
I feel frustrated when others omit hand hygiene (attitude)	380(83.9)	73(16.1)	244(64.2)	136(35.8)	<0.001

**Notes.**

N number of study participants % percentage

Females had significantly better knowledge than males regarding several aspects of the kind of HH actions for preventing germ transmission to patients and the health-care workers; alcohol-based hand rub and hand-washing with soap and water (Table 2A).

Interestingly, only 135 (29.4%) of the participants had correct knowledge about the least time required for alcohol-based hand rubbing to eradicate the majority of the microbes on hands. There was no significant difference found between males and females about this item (Table 2B).

Females had better knowledge than males regarding the type of HH technique needed before palpation of the abdomen [177(54%) Vs. 151(46%); *P* < 0.002], before an injection [175(54.5%) Vs. 146(45.5%); *P* < 0.007], after emptying a bedpan [207(64.7%) Vs 113(35.3%); *P* < 0.001], following discarding examination gloves [256(60.4% Vs. 168(39.6%); *P* < 0.001] and after exposure to blood [200(64.1%) Vs. 112(35.%); *P* < 0.001) (Table 2B).

Besides, there was no significant variance between both genders’ knowledge regarding the avoidance of factor that is linked with the augmented probability of colonization of hands with damaging germs except in the regular use of hand cream [244(62.7) Vs. 145(37.3%; *P* < 0.001]. Females also had a significant better attitude towards the importance of HH than males [240(62.5%) Vs. 144(37.5%); <0.001] (Table 2B).

## Discussion

Our study results show that there is a lack of awareness regarding HH among study participants.

In the present study, more than half (64.2%) of the medical students and interns received formal training in hand hygiene. The researcher didn’t provide any training to the respondents. However, a few of them participated in a workshop on HH and others attended awareness sessions. Nair et al. (2014) reported that 79% of students had formal training in HH whereas Kamble et al. (2016) reported 85.4% of medical students had formal training in HH practices. Hence, in our study, the knowledge of HH is not as good as other studies.

Alcohol-based hand rubbing has a fundamental role in preventing the transmission of many organisms (Todd et al., 2010). Our study showed that 66.9% of participants used alcohol -based hand rub routinely, that is similar to another study (Modi et al., 2017).

More than half of the participants (56.1%) answered correctly that the main route of germ transmission is uncleaned hands (rendering them infected) of healthcare workers. Similarly, 40% and 75% of medical students were able to acknowledge this fact in two other studies (Brosio et al., 2017; Nair et al., 2014). Additionally, two previous studies from the SA reported that 41%, and 55% of students answered correctly to the question (Amin et al., 2013; El Bingawi et al., 2017).

Among our participants, only one-fourth of the students answered correctly that the frequent source of healthcare-associated infections are germ related to the patient. While previously, a study by Amin et al. (2013) reported that 41% of medical students had correct knowledge.

Knowledge about HH before touching the patient was 89.2% and after body fluid exposure was 74.4%. Hamadah et al., stated that 72% of medical students knew about HH before getting in contact with patients and 68% after contact of fluid secretion (Hamadah et al., 2015), while a Sri Lankan study asserted that 93% of medical students knew about HH before touching a patient and 80% after contact of fluid secretion (Ariyaratne et al., 2013).

In the present study, the majority of participants responded correctly about the importance of HH for a clean/aseptic practice, following interaction with body fluid, before and after touching the patient, and exposure to immediate surroundings of a patient. Our results are similar to that of Kamble et al. (2016).

About half of the participants (55.4%) agreed that hand rubbing is more rapid than washing and 49.2% approved that hand washing dried the skin better than hand rubbing. Nair et al. (2014) reported that 69% and 30.2% of medical students were aware of this fact.

According to the guidelines of the CDC (2015), hand washing with water and regular soap is the best available method to limit germ numbers. Hand washing is especially important in highly contaminated or greasy hands (Edmonds et al., 2010), and it can eradicate the spread of particular types of bacteria like *Clostridium difficile* (Oughton et al., 2009).

Two-third of the participants agreed that hand rubbing is less effective than washing and 30% agreed that hand washing and hand rubbing should not be performed in sequence. Whereas Thakker & Jadhav (2015) reported 66% and 45% of medical students were aware of both these facts.

Awareness of the effective time and appropriate use of alcohol-based hand rubbing is very important in preventing the spread of microbes (Kampf et al., 2010). Only 30% of participants revealed the appropriate time required for alcohol-based hand rubbing to eradicate most microorganisms on the hands, while Amin et al. (2013) reported 42% of the knowledge of this fact.

Differentiation between the use of hand rubbing and hand washing in different conditions was deficient among the participants, and 72.4%, 70.9% and 46.4% of participants declared that rubbing was the method of HH before palpation of the abdomen, before injection and after making a patient’s bed. However, 70.6% and 68.9% of participants reported that hand washing was the method of hygiene after emptying bedpan and after contact with blood.

Participants reported that wearing jewelry (73.5%), skin injuries (74.2%) and artificial fingernails (20.8%) should be avoided, as they are related with the augmented probability of colonization of hands with detrimental germs. Also, they agreed that consistent application of hand cream (85.9%) does not increase that risk. Our results are more or less similar to that of Modi et al. (2017).

In this study, female students had a significantly better knowledge than males regarding the kind of HH actions for preventing germ transmission to patients and the health-care workers: alcohol-based hand rub and hand-washing with soap and water. Females had better significant knowledge than males regarding the type of HH technique needed before palpation of the abdomen, before injection, after emptying a bedpan, following discarding examination gloves, and after exposure to blood. Females also had a better significant attitude towards the importance of HH than males. We could not find any specific reason for better knowledge and attitude regarding HH among women as compared to men. It may be assumed that women are usually more cautious about overall hygiene practices including HH. Similar to our results, a study from Pakistan reported that the females had better HH perception (Zil-E-Ali et al., 2017).

In our study, participants reported positive attitudes towards HH. Majority of the participants (84.8%) felt guilty when they omitted HH and 83.9% felt frustrated when others omitted it. This positive attitude is a good base to improve the right knowledge and practice of HH in the future.

There is a discrepancy between awareness of the HH and its compliance with 58% missed HH as they forgot to do it. This means a lack of regular practice of HH instructions. The results of our study indicate that respondents’ knowledge was not good in spite of the fact that all participants were medical students and interns. Not only ours, but several other studies have documented less compliance on HH principles. It is a serious issue and exigent steps are needed to improve HH compliance among health professionals’ as there are several ways to improve the awareness and compliance of HH. It is important to implement and update formal training programs on HH for medical students, taking into consideration WHO and CDC guidelines. Furthermore, the behavior of students is influenced by their mentor’s attitude at the bedside, and if the mentor is not following the established HH rules, then there is a lot of probability that in future, students will do the same. Previous studies stated that students’ response was mainly influenced by their role models’ behaviors towards HH (Anwar et al., 2009; Jang et al., 2010).

Hand hygiene is one of the most effective procedures against hospital-acquired infections (Kampf & Kramer, 2004). Therefore, we suggest that the senior physicians should take care of the HH practices regularly so that their juniors can learn and make it a compulsory part of their daily practice. It is also important to teach HH to the undergraduate level in the curriculum or by organizing workshops on a regular basis. A recent study reported that HCPs perceived HH as “an added extra rather than an integral part of the process” (Cresswell & Monrouxe, 2018).

There are a few limitations to our survey. This is a questionnaire-based study, so the authenticity of the responses cannot be ensured. The respondents’ personal biases cannot be ignored. It is a single center study so it is quite possible that in other medical colleges of Saudi Arabia, the students’ knowledge may be better or vice versa.

## Conclusions

Our results show that majority of the participants’ knowledge regarding HH is not up to the mark, however, female students had a better knowledge as compared to male students.

The medical students and interns’ knowledge and positive attitude towards HH can play a pivotal role in preventing HCPs associated infections and it would overall decrease the infection-related burden on the hospital and governmental budgets. It is suggested that multi-dimensional plans are required to change this low compliance to a higher rate and moreover, the implementation of the WHO and CDC guidelines need to be followed properly. The basic and clinical sciences teachers should make them aware about the full compliance of the HH and adverse effects of non-compliance on themselves and patients. The clinical teaching staff should also observe medical students practices for HH during medical ward rotations, as it will probably enhance the practice of HH.

##  Supplemental Information

10.7717/peerj.6823/supp-1 DataSet S1 Raw data of the study participants This data is showing the responses of the participants related to hand hygiene questionnaire.

## Additional Information and Declarations

### Data Availability

The following information was supplied regarding data availability: The raw data are available in Dataset S1.
